# Portland Town Free Dispensary

**Published:** 1903-06-06

**Authors:** 


### HOSPITAL ADMINISTRATION.

### CONSTRUCTION AND ECONOMICS.

### PORTLAND TOWN FREE DISPENSARY.

#### THE MEDICAL ATTENDANCE OF LONDONERS.

The annual meeting of the Portland Town Free Dispensary was held on May 28th at the De Walden Institute, Charlbert Street, St. John's Wood. In moving the adoption of the report and balance-sheet Sir Henry Burdett, who was in the chair, said the dispensaries of London, which were amongst the oldest institutions in the metropolis, had done for more than a century most useful work, and they had the great merit that they had never departed from the principles upon which they were originally founded—that they should be maintained and managed for the poor—and it was to their credit that they stood to-day alone amongst the medical institutions of the metropolis as institutions which in practice were not abused. This fact probably pointed the direction in which the necessary reform must come in regard to the question of free medical attendance for Londoners. One great merit of the dispensary was that whereas an out-patient in a London hospital, according to the statistics and figures of the Hospital Sunday Fund, cost 2s., the cost for one of the out-patients at the dispensary was 9d. at the outside, and probably less. They had, too, a most useful and special branch of work—the home visiting of cases—which not only provided in the best way by the medical attendance of the sick in their own homes, but ensured to those homes a maximum of sanitary attention, and, where necessary, the introduction of hygienic improvements through the local health authorities which would certainly be delayed and might never come, if it were not for the attention called to them by the visiting medical officer. Last year 3,000 patients were seen at this dispensary and 750 at their own homes, and if anyone would come forward with another £100 a year, these numbers could be doubled. He hoped that whatever result might come out of the meeting that day, it would not longer be possible to say of the large number of well-to-do people round that district that they were indifferent to the claims of this institution, or, thanks to the press, that they were ignorant of those claims.

Dealing next with the question of the medical attendance of Londoners from the point of view of medical education, Sir Henry said one of the difficulties which the hospitals have had in financing themselves had arisen from the attacks made by the anti-vivisectionists. If it were not for this question of medical education the anti-vivisectionist would have no case. In Sir Henry's view it was, however, manifestly wrong that any hospital committee should devote to the purposes of medical education any portion of funds subscribed by the public for the relief of the sick. Doctors were drawn from the same classes of the population as the members of other professions; were the sons of relatively well-to-do people, who were absolutely as prepared to pay an adequate sum for the medical equipment of their sons, if they entered the medical profession, as they did now cheerfully pay adequate sums for their equipment for other professions. How was it then that the fees charged were not adequate to give the necessary facilities for medical education, and that the conductors of the medical schools came back upon the hospital committees and asked them to divert a portion of the free gifts of the public which ought to be expended wholly upon the relief of the sick? The answer was that in London there was no great medical school as a centre. The system of medical education was in a state of disorder. If there were one big medical school for all the students of various hospitals it would then be possible to get our system of medical education into order, and no more would be heard of these attacks of the anti-vivisectionists. He could only say that he did most heartily hope that the public would combine as one man by setting their face absolutely against this perversion of the funds of the hospital to the medical school. There was great need of a reconsideration of the present position of medical education in the metropolis of the Empire, and so London might be capable of being compared favourably with other large Continental and American cities.

Looking at the question from the point of view of the theory of attendance the rich were well provided for, for there were in London to-day upwards of 6,000 medical men to attend to a population of something like $4\frac{1}{2}$ millions; whilst the poor had dispensaries like the Portland Town Free Dispensary, and they would be well advised to avail themselves more frequently of these institutions; but in practice the tendency was to go in larger and larger numbers to the hospitals. Speaking twenty years ago on this question Sir Henry had said that when one person in three or one person in four went to the hospitals there was something wrong. But this question of seeking hospital relief had since gone on extending to all classes to the great injury and robbery of the medical profession and to the great injury of the moral fibre of the people too. To-day the growth in hospital attendance was so great that it exceeded, or at any rate equalled, the actual growth in the population of London. On a moderate computation the population increased 60,000 every year, and the hospitals positively had to attend every year to something like 60,000 additional patients. Taking a period of seven years, in 1895 all the hospitals of London relieved 1,753,611, and in 1902 they relieved 2,098,905 patients, showing an increase in seven years of 345,294 patients receiving free medical relief, so that practically one out of every two Londoners in some form or shape got free medical relief from some institution. Now that was a very grave abuse, and it was a position which London should take to heart, as well as the hospital authorities. The result of this pressure on the hospitals was that the average cost of an occupied bed in a big hospital like the London or Guy's Hospital had gone up in ten years by 25 per cent., so that whereas a bed used to cost £81 it now cost £101 or £102 per annum to maintain; and in the case of the London Hospital the increase in the cost of salaries alone was 33 per cent. beyond what it was ten years ago, and at Guy's Hospital the growth of expenditure on salaries was 25 per cent.

The hospital patient had many advantages over the patients of the consultants, for at the hospital, although he might have to wait a long time, there was the absolute certainty that in the end he would ultimately be seen, examined, and prescribed for, which was not always the case at the consultant's, who might be called away before he could see all his patients. Further, a great deal of money was spent by the hospitals in making them hygienically perfect, so that the patients ran no avoidable risk of infection whilst waiting their turn to be seen. This was far from being the case with some of the West End waiting-rooms, where full fees were paid by the patients.

Again the general body of the medical profession had to suffer competition both from the hospitals and the consultants. But the complaint was in some parts of London that the general practitioner had not the opportunities and facilities of keeping up with his profession, and therefore the hospitals were necessarily the best thing for the patients. It was, however, very unjust to any general practitioner to make any such claim in justification of sponging on the hospitals, because every general practitioner had the opportunity of keeping in touch with his work by going to the hospitals and seeing the latest developments in medical knowledge through the post-graduate classes. This being so, if people would only realise what was best for themselves, both morally and physically, they would certainly never go as out-patients until they had first of all seen what their local doctor could do for them at home. This led to the question, could matters remain as at present? Was it possible to allow the hospitals to encourage the people to come to them in ever-increasing numbers, because it was urged we must not keep anybody away or we may lose an interesting case from a clinical point of view. He said emphatically matters could not so remain, because underlying all this question was the financial stability of the voluntary hospital system. To-day the voluntary hospitals were, for all practical purposes, financed. Their total expenditure for 1902 was £1,161,000, and they had a revenue of £1,158,000, so that the whole deficiency of all the medical institutions was only £3,000. The reason that the public were allowed to suppose that the voluntary hospitals were so impecunious, that they were constantly in peril of going on the rates, was because they were misled by the fact, that many people in stating the income of a charitable institution ignored legacies. Yet the legacies were a constant source of revenue and ought to be so treated, for the average yearly amount raised from legacies was a constant factor which had a tendency to increase in these days. Matters could not remain as they were, partly on account of the cost, as it was quite impossible to suppose that we had in these days reached finality in the cost per bed. In practice it came to this: hospitals were great educational centres, and they ought to be so economically administered that they were examples of the maximum efficiency at the least cost; but the tendency to-day was to show them as examples of efficiency at the maximum cost. The present system of unlimited free medical attendance was a great mistake and a great danger.

The remedy existed in London to-day; it had been applied with marked effect, and it was one which the hospitals could adopt without any change in their constitutions or any great delay. In the first place, every hospital should arrange to treat only urgent cases. Every urgent medical and surgical case should receive attention at the hospital. The out-patient department as at present organised should be abolished altogether. Then the hospitals should see and examine every patient who was sent to them by a doctor. Every member of the medical profession should have the advantage of co-operating with the hospitals and so secure that there should be no abuse of hospitals such as existed to-day. When the patients sent by the doctors had been seen by the medical staff at the hospitals, every such case found to be suitable should be handed on to the provident dispensary, *i.e.*, to the reformed out-patient department of each hospital. This was a very simple system, but it cut at the root of the present abuses. It would please and help the people and the medical profession, and would give courage to the generous souls who had laboured so hard to finance the hospitals. The latter would then see that there was at least an attempt at finality on the part of hospital managers in regard to the ever-growing expenditure, and they might then hope that under such a system would soon come increased efficiency. At Battersea the working classes and the doctors had united with the Bolingbroke Hospital, and they did there carry out a scheme of out-patient relief on the lines just explained. The system worked admirably for everybody; there was nothing that could be fairly urged against it, and it was a very great success. During the last year 36,564 patients had been treated at Battersea in the provident out-patient department. That is more than four times as many patients as those treated by all the other dispensaries in the district. This showed that this system was much more widely popular than the system of free medical relief. It ought, therefore, to have the candid and immediate attention of all those in charge of our hospitals, who wished to see these institutions efficient.

